# Intravitreal Injections and Face Masks: Endophthalmitis Risk Before and During the COVID-19 Pandemic

**DOI:** 10.18502/jovr.v18i3.13776

**Published:** 2023-07-28

**Authors:** Saeed Karimi, Homayoun Nikkhah, Amir Mohammadzadeh, Alireza Ramezani, Iman Ansari, Hosein Nouri, Seyed-Hossein Abtahi

**Affiliations:** ^1^Ophthalmic Research Center, Research Institute for Ophthalmology and Vision Science, Shahid Beheshti University of Medical Sciences, Tehran, Iran; ^2^Department of Ophthalmology, Torfeh Medical Center, Shahid Beheshti University of Medical Sciences, Tehran, Iran; ^3^Clinical Research Development Unit of Torfeh Medical Center, Shahid Beheshti University of Medical Sciences, Tehran, Iran; ^4^Ophthalmic Epidemiology Research Center, Research Institute for Ophthalmology and Vision Science, Shahid Beheshti University of Medical Sciences, Tehran, Iran; ^5^School of Medicine, Isfahan University of Medical Sciences, Isfahan, Iran

**Keywords:** COVID-19, Endophthalmitis, Face Mask, Intravitreal Injection, Infection

## Abstract

**Purpose:**

To assess the added risk of acute endophthalmitis after intravitreal injections associated with the widespread use of face masks during the COVID-19 pandemic.

**Methods:**

In this retrospective, single-center study, records of patients with acute endophthalmitis following intravitreal bevacizumab (IVB) injections during the pre-COVID era—that is, March 1 ${\;}^{\text{st}}$ , 2013 to October 31 ${\;}^{\text{st}}$ , 2019 —and the COVID-19 era—that is, March 1 ${\;}^{\text{st}}$ , 2020 to April 1 ${\;}^{\text{st}}$ , 2021 —were reviewed and compared.

**Results:**

A total of 28,085 IVB injections were performed during the pre-COVID era; nine eyes of nine patients developed acute post-IVB endophthalmitis in this era, giving an overall incidence of 0.032% (3.2 in 10,000 injections). In the COVID era, 10,717 IVB injections were performed; four eyes of four patients developed acute post-IVB endophthalmitis in this era, giving an overall incidence of 0.037% (3.7 in 10,000 injections). The incidences of post-IVB endophthalmitis during these two eras were not statistically significantly different (*P* = 0.779).

**Conclusion:**

Face masking protocols seem unlikely to impose any additional risk of post-IVB endophthalmitis.

##  INTRODUCTION

The importance of intravitreal injections, especially intravitreal anti-vascular endothelial growth factor (anti-VEGF) injections, has increased in recent years due to their widespread use in managing retinal diseases such as diabetic macular edema (DME) and neovascular age-related macular degeneration (nAMD).^[1]^ Along with its therapeutic benefits, the intravitreal delivery route carries the risk of some side effects and complications, including subconjunctival hemorrhage, uveitis, retinal tear or detachment, and endophthalmitis.^[2,3]^ Endophthalmitis is a severe, sight-threatening complication of intravitreal injections; among its known risk factors are diabetes mellitus (DM), blebs, blepharitis, etc.^[3,4]^ Adherence to standard injection protocol can significantly limit the incidence of post-intravitreal injection endophthalmitis.^[3,5]^ Alterations in the former may give rise to changes in the latter. The probable impacts of health protocol amendments during the COVID-19 pandemic, for example, mask-wearing mandates for patients and staff^[5]^ and increasing application of hand disinfectant solutions in hospitals^[6,7]^ during the COVID-19 pandemic^[8,9]^ on endophthalmitis incidence after intravitreal injection of anti-VEGF drugs are under investigation; however, the findings have been inconsistent. While some have suggested an additional risk of endophthalmitis with patients wearing masks during the sessions—theoretically, through an upward direction of exhaled vapors toward the periocular area, increasing the risk of infectious complications,^[10]^ others have found no increase in the overall risk of endophthalmitis, attributable to using face.^[11]^

In the present study, we investigated the effect of these changes in health protocols during the COVID pandemic on the incidence of post-intravitreal injections endophthalmitis and compared the endophthalmitis rate during the COVID pandemic with the rate at which it occurred during the pre-COVID era. Intravitreal bevacizumab (IVB) was the most commonly used intravitreal anti-VEGF in the center where the study was conducted; thus, the term “IVB” will be used instead of intravitreal anti-VEGF injection throughout this paper.

##  METHODS

In this retrospective, single-center cohort study, the electronic medical records of the Torfe Eye Hospital, affiliated with the Shahid Beheshti University of Medical Sciences, were accessed and queried for all cases of acute endophthalmitis following IVB injections, performed during two distinct periods—March 1 ${\;}^{\text{st}}$ , 2013 to October 31 ${\;}^{\text{st}}$ , 2019 and March 1 ${\;}^{\text{st}}$ , 2020 to April 1 ${\;}^{\text{st}}$ , 2021. The former period is from now on referred to as the “pre-COVID era”, and the latter as the “COVID era,” constituting approximately 80 and 12 months, respectively.

The study protocol was approved by the local ethics committee and adhered to the tenets of the Declaration of Helsinki (ethics code: IR.SBMU.MSP.REC.1396.737). The records of all patients who had undergone IVB injections during those periods were extracted from the hospital data archives using the respective current procedural terminology (CPT) code:67028. Subsequently, among those records, all cases with acute post-IVB endophthalmitis were identified and extracted using the International Classification of the Disease-10 (ICD-10) codes of endophthalmitis:H44.0-H44.1. The acquired information included demographic data, injection indications, treatments and outcomes, bacterial smear and culture results, best-corrected visual acuity scores (BCVA) before the injection and after the onset of endophthalmitis symptoms, and three months after the treatment.

Post-IVB endophthalmitis cases were excluded if (I) their bevacizumab administration was part of another surgical procedure, (II) they had any other intraocular procedures during the last six weeks before the IVB injection, and (III) their acute endophthalmitis was secondary to other causes such as trauma or post-cataract surgery. Acute endophthalmitis after IVB was defined as progressive inflammation in the vitreous cavity with or without inflammation of the anterior chamber, occurring within six weeks after the injection. The diagnosis was confirmed by a vitreoretinal surgeon.

All injections at the Torfe Eye Hospital were performed in the operating room under strict aseptic conditions. In the pre-COVID era, no face masks were used by patients during the procedure; however, during the COVID era patients had to wear face masks while being injected, holding them under their noses. Before the procedure, physicians scrubbed their hands and wore face masks and sterile gloves. Topical tetracaine 0.5% drops were used to achieve local anesthesia. After cleaning the skin around the eye with a solution of povidone-iodine 10% and instilling a single drop of povidone-iodine 5% in the cul-de-sac, followed by setting an ophthalmic drape – without adhesive bands – a sterile lid speculum was placed. The rubber covering of the bevacizumab vial was wiped with cotton soaked in 5% betadine; 1.25 mg/0.05 ml Avastin (Genentech, CA, USA, 100 mg/4 ml vial; for injections before November 2018)or Stivant (CinnaGen, Iran, 100 mg/4 ml vial; for injections in November 2018 and thereafter) was drawn into an insulin syringe for each injection—Stivant, a biosimilar for Avastin, became available to use in Torfe Medical Center from November 2018. The needle was then changed, and a 30-gauge needle was used for injection at 3–4 mm posterior to the limbus. Topical antibiotic eye drops were prescribed for three days after injection.^[12]^ Except for the patients' mask-wearing mandate, all mentioned measures were similar to the pre-COVID era.

In the case of post-IVB endophthalmitis, an immediate anterior chamber and vitreous tap was performed for all patients, followed by intravitreal injection of vancomycin (1 mg) and ceftazidime (2.25 mg). Early standard three-port pars plana vitrectomy (PPV) was performed within 24 hr of diagnosis. Fortified antibiotic eye drops (vancomycin and ceftazidime) and systemic intravenous vancomycin and ceftazidime were initiated for all patients—systemic intravenous antibiotic therapy preceded all other therapeutic measures and was initiated immediately after the patients' admission. Oral prednisolone 1 mg/kg was prescribed 24 hr after PPV and continued for 10 days.

Finally, to investigate the effect of the pandemic-associated adjustments in health protocols on the incidence of this complication, data from the two periods, that is, the pre-COVID and the COVID eras, were compared and analyzed. Normal continuous variables were described as mean and standard deviation, and qualitative variables as frequency and percentage. Chi-square test, Wilcoxon signed-rank test, and Kruskal–Wallis test were used to evaluate visual acuity changes in relation to other variables. The Fisher's exact test was applied when comparing variables from the pre-COVID era with those from the COVID era. A *P*-value of < 0.05 was considered statistically significant. Data was gathered and analyzed using IBM SPSS v.23.0. for Windows.

##  RESULTS

### Data from the Pre-COVID Era

During the pre-COVID era, 28,085 IVB injections were performed at the Torfe Eye Hospital. Nine eyes of nine patients developed acute post-IVB endophthalmitis, giving an overall incidence of 0.032% (3.2 in 10,000 injections)—no cluster pattern was observed in incident endophthalmitis episodes. The patients' mean ( ± SD) age was 63.78 years ( ± 13.8; range, 44–89). Six patients (66.7%) were female. The indications for IVB injections were DME in four eyes (44.5%), vitreous hemorrhage due to proliferative diabetic retinopathy (PDR) in three eyes (33.3%), and neovascular AMD (nAMD) in two eyes (22.2%). Considering all IVB injections in the pre-COVID era (28,085 injections), the incidences of post-IVB endophthalmitis were 0.014%, 0.010%, and 0.007% in PDR, nAMD, and DME patients, respectively (*P* > 0.05). In the pre-COVID era, the mean ( ± SD) time between IVB injections and endophthalmitis presentation was 2.77 days ( ± 1.25; range, 1–6). Table 1 presents detailed information on the nine patients who developed post-IVB endophthalmitis in the pre-COVID era.

### Data from the COVID Era

During the COVID era, 10,717 IVB injections were performed at the Torfe Eye Hospital. Four eyes of four patients developed acute post-IVB endophthalmitis giving an overall incidence of 0.037% (3.7 in 10,000 injections), with no cluster pattern of incidence. The patients' mean ( ± SD) age was 63.25 years ( ± 6.5, range, 55–69). Among the four, only one was male. The indication for IVB injections was DME in three eyes (75%) and nAMD in one eye (25%). The mean ( ± SD) time between IVB injections and the endophthalmitis presentation was 2.75 days ( ± 1.71; range, 1–5). Table 2 shows detailed information on patients with post-IVB endophthalmitis in the COVID era. No significant difference was observed in the incidence of endophthalmitis in pre-COVID and COVID eras (0.032% vs 0.037%; *P* = 0.779).

**Table 1:** Demographic, clinical, and culture information of patients with post-IVB endophthalmitis during the pre-COVID era.


**Case No.**	**Sex**	**Age**	**Indication**	**Days to presentation**	<@orange**Pre-injection BCVA**		<@orange**Presentation BCVA**		<@orange**Final BCVA**		**Culture**
			Snellen	LogMAR	Snellen	LogMAR	Snellen	LogMAR	
1	F	60	PDR	4	1/10	1	HM	2.6	CF1m	1.79	No growth
2	M	79	CNV	1	CF3m	1.31	LP	2.7	HM	2.6	No growth
3	F	86	PDR	6	2/10	0.7	NLP	3	NLP	3	*Staphyloccous epidermidis*
4	M	65	DME	2	3/10	0.52	CF1m	1.79	2/10	0.7	No growth
5	F	44	DME	2	CF2m	1.48	HM	2.6	CF2.5m	1.39	No growth
6	F	58	CNV	2	2/10	0.7	HM	2.6	1/10	1	*Staphyloccous epidermidis*
7	F	88	DME	2	CF3m	1.31	CF1m	1.79	CF2.5m	1.39	No growth
8	F	54	DME	4	1/10	1	LP	2.7	NLP	3	*Staphyloccous epidermidis*
9	M	50	PDR	2	HM	2.6	LP	2.7	HM	2.6	No growth
Mean ± SD		<@63.78 ± 13.8		2.78 ± 1.56	1.18 ± 0.62	2.5 ± 0.42	1.94 ± 0.88	
	
	
BCVA, best-corrected visual acuity; CF, counting finger; CNV, choroidal neovascularization; DME, diabetic macular edema; F, female; HM, hand motion; LogMAR, logarithm of minimum angle of resolution; LP, light perception; M, male; NLP, no light perception; PDR, proliferative diabetic retinopathy; SD, standard deviation											

**Table 2:** Demographic, clinical, and culture information of patients with post-IVB endophthalmitis during the COVID era.


**Case No.**	**Sex**	**Age**	**Indication**	**Days to presentation**	<@orange**Pre-injection BCVA**		<@orange**Presentation BCVA**		<@orange**Final BCVA**		**Culture**
			Snellen	LogMAR	Snellen	LogMAR	Snellen	LogMAR	
1	F	69	DME	1	4/10	0.4	1/10	1	4/10	0.4	No growth
2	F	55	DME	5	CF1m	1.79	HM	2.6	CF2m	1.48	No growth
3	F	61	DME	2	CF3m	1.31	HM	2.6	2/10	0.7	No growth
4	M	68	AMD	3	2/10	0.7	HM	2.6	CF3m	1.31	No growth
Mean ± SD		<@63.25 ( ± 6.5)		2.75 ± 1.71	1.05 ± 0.62	<@2.20 ± 0.80		0.97 ± 0.51	
	
	
AMD, age-related macular degeneration; BCVA, best-corrected visual acuity; CF, counting finger; DME, diabetic macular edema; F, female; HM, hand motion; LogMAR, logarithm of minimum angle of resolution; M, male; SD, standard deviation											

### Culture Results

Among the nine endophthalmitis cases documented during the pre-COVID era, six (66.7%) showed negative culture results, while three (33.3%) showed staphylococcus epidermidis growth; one eye developed phthisis bulbi (11.1%). Culture results were negative for all four endophthalmitis cases in the COVID era.

##  DISCUSSION

The present study determined a post-IVB endophthalmitis rate of 0.032% (3.2 in 10,000 injections) in the pre-COVID era and 0.037% (3.7 in 10,000 injections) in the COVID era; our results are consistent with the ranges reported in previous studies.^[13]^

Post-IVB endophthalmitis is a serious complication. Based on previous studies, factors affecting the development of endophthalmitis following intravitreal injections can be divided into clinical and technical categories. Clinical factors that can increase the risk of endophthalmitis include DM, older age, and blepharitis. DM and older age have also been associated with immunosuppression and increased susceptibility to infection. ${\;}^{\left[14,15\right]}$ Among technical factors is the type of surgical equipment used, as well as how well health protocols are observed. It is worth mentioning that our university hospital is an evolving medical center with increasing referral rates over the past few years, which explains why the total number of cases referred for receiving IVB during the pre-COVID era ( ∼ 6 years) was only 2.8 times than that during the COVID-era ( ∼ 1 year). Moreover, the change in the medication used (i.e., Avastin until late 2018 vs Stivant after that) is unlikely to have had any confounding effect on the results because endophthalmitis cases were not in clusters, and incidence was not changed between the two eras. Furthermore, the small number of positive-culture cases precludes an accurate comparison and a meaningful, relevant discussion.

Overall, we found that altered health protocols during the COVID pandemic had no statistically significant effect on the incidence of post-IVB endophthalmitis. A few studies have evaluated the effect of universal face mask-wearing and other pandemic health protocols on the rate of endophthalmitis after intravitreal injections. ${\;}^{\left[11,16\right]}$ A multicenter and retrospective study has reported that universal face mask use during intravitreal injections did not increase the risk of developing presumed endophthalmitis, but it was associated with a lower rate of culture-positive endophthalmitis.^[16]^ In another study by Patel et al, face mask use by physician did not influence the risk of post-injection endophthalmitis as compared to a no-talking policy.^[11]^. It was hypothesized that facial mask fitting by surgeons could effectively affect bacterial transmission and the risk of post-intravitreal injection endophthalmitis.^[17]^ In a study by Hadayer et al, it was emphasized that patients who wear face masks during intravitreal injections might be at a higher risk of endophthalmitis; hence, face masks with proper fitting, or taping the upper edges of the face masks with a medical adhesive tape, or using an adhesive surgical drape around the injected eye were recommended.^[18]^ Another study by simulation of intravitreal injections concluded that adding tape to the superior portion of the patient's face mask reduces bacterial dispersion during intravitreal injections. Also, bacterial dispersion was not different compared to wearing N95 masks.^[19]^ Another study suggested that securing the superior portion of the patient's face mask with tape may reduce bacterial dispersion or air particles toward the eye.^[20]^ However, it has been reported that this measure has no effect on endophthalmitis risk in patients undergoing IVB injections.^[16]^

Some limitations apply to the present study. Given the retrospective nature of this study, potential errors in data registering in the hospital records could have been present; however, restrictive measures were taken to minimize such errors. In addition, with the ongoing pandemic, a decline in the number of patients—especially diabetic patients, many of whom suffer from other underlying comorbidities—referring to hospitals^[21]^ is a limitation that applies to many hospital-based studies; this limitation is more prominent in cohort studies evaluating the incidence of an uncommon complication, such as ours. Furthermore, results from this study may be inferred only to injection settings similar to that of this study; that is, office-based injections with variable degrees of adherence to standard injection protocols may present different complication incidences.

In summary, the present study showed that the incidence of post-IVB endophthalmitis in the COVID era was not significantly different from the pre-pandemic era. Regardless of the pandemic-related alterations in health protocols adopted—mandatory face masking, in particular—endophthalmitis remains a rare complication after intravitreal injections. The time interval between the IVB injection and presentation of endophthalmitis is relatively short; prompt treatment with immediate intravitreal antibiotics and early PPV are vital in maximizing positive treatment outcomes.

##  Financial Support and Sponsorship

None.

##  Conflicts of Interest

None.
